# Prevention of pressure ulcers in spinal cord injury

**Published:** 2012-11

**Authors:** Fereshteh Karam Pazhouh, Nasrin Parvaz, Hamid Reza Saeidi Borojeni, Tayebeh Mahvar, Zhaleh Alimoradi

**Affiliations:** ^*a*^Department of Neurosurgery, Teleghani Hospital, Kermanshah University of Medical Sciences, Kermanshah, Iran.; ^*b*^Vice Chancellery for Research and Technology, Kermanshah University of Medical Sciences, Kermanshah, Iran.

## Abstract

Pressure ulcers are a lifelong, serious complication of spinal cord injury. They have the potential to interfere with physical, psychological, and social well-being and to impact overall quality of life.

Prevention Strategies:

1. Implementing pressure ulcer prevention strategies as part of the comprehensive management of acute SCI and reviewing all aspects of risk when determining prevention strategies.

- Avoiding prolonged positional immobilization whenever possible.

2. Conducting daily comprehensive visual and tactile skin inspections, with particular attention to the regions most vulnerable to pressure ulcer development.

3. Turning or repositioning individuals with SCI initially every 2 hours in the acute and rehabilitation phases if the medical condition allows.

4. Evaluating the individual and his/her support environment for optimal maintenance of skin integrity.

5. Providing an individually prescribed wheelchair and pressure-reducing seating system.

6. Implementing an ongoing exercise regimen for the medically stable SCI individual to promote maintenance of skin integrity, improve cardiovascular e, and prevent fatigue and deconditioning.

7. Providing individuals with SCI, their families, significant others, on effective strategies for the prevention and treatment of pressure ulcers.

8. Assessing nutritional status of all SCI individuals on admission and as needed, based on medical status, including:

9. Providing adequate nutritional intake to meet the individual’s needs, especially:

- Calories (or Energy)

- Protein

- Micronutrients (zinc, vitamin C, vitamin A, and vitamin E)

- Fluids

10. Implementing aggressive nutritional support measures if dietary intake is inadequate or if an individual is nutritionally compromised.

Treatment:

Nonsurgical:

- Cleansing

- Debridement

- Dressings

- Electrical stimulation

- Reassessment

Surgical:

- Excising of ulcer, surrounding scar, bursa, soft tissue calcification, and underlying necrotic or infected bone

- Filling dead space, enhancing vascularity of the healing wound, and distributing pressure off the bone

- Resurfacing with a large regional pedicle flap, with suture line away from the area of direct pressure, and one that does not encroach on adjacent flap territories

- Preserving options for future potential break-downs

**Keywords:**

Prevention, Pressure ulcers, Spinal Cord Injury


					Volume 4, Suppl. 1 Nov 2012
Publisher: Kermanshah University of Medical Sciences
URL: http://www.jivresearch.org
Copyright:
Congress of Iranian Neurosurgeons, 14-16 November 2012, Kermanshah, Iran
Paper No. 90
Prevention of pressure ulcers in spinal cord injury
Fereshteh Karam Pazhouh a
, Nasrin Parvaz a
, Hamid Reza Saeidi Borojeni a,*
, Tayebeh Mahvar a
,
Zhaleh Alimoradi b
a
Department of Neurosurgery, Teleghani Hospital, Kermanshah University of Medical Sciences, Kermanshah, Iran.
b
Vice Chancellery for Research and Technology, Kermanshah University of Medical Sciences, Kermanshah, Iran.
Abstract:
Pressure ulcers are a lifelong, serious complication of spinal cord injury. They have the potential to interfere with
physical, psychological, and social well-being and to impact overall quality of life.
Prevention Strategies
1. Implementing pressure ulcer prevention strategies as part of the comprehensive management of acute SCI and
reviewing all aspects of risk when determining prevention strategies.
- Avoiding prolonged positional immobilization whenever possible.
2. Conducting daily comprehensive visual and tactile skin inspections, with particular attention to the regions most
vulnerable to pressure ulcer development.
3. Turning or repositioning individuals with SCI initially every 2 hours in the acute and rehabilitation phases if the
medical condition allows.
4. Evaluating the individual and his/her support environment for optimal maintenance of skin integrity.
5. Providing an individually prescribed wheelchair and pressure-reducing seating system.
6. Implementing an ongoing exercise regimen for the medically stable SCI individual to promote maintenance of skin
integrity, improve cardiovascular e, and prevent fatigue and deconditioning.
7. Providing individuals with SCI, their families, significant others, on effective strategies for the prevention and
treatment of pressure ulcers.
8. Assessing nutritional status of all SCI individuals on admission and as needed, based on medical status, including:
9. Providing adequate nutritional intake to meet the individual's needs, especially:
- Calories (or Energy)
- Protein
- Micronutrients (zinc, vitamin C, vitamin A, and vitamin E)
- Fluids
10. Implementing aggressive nutritional support measures if dietary intake is inadequate or if an individual is
nutritionally compromised.
Treatment
Nonsurgical
- Cleansing
- Debridement
- Dressings
- Electrical stimulation
- Reassessment
Surgical
- Excising of ulcer, surrounding scar, bursa, soft tissue calcification, and underlying necrotic or infected bone
- Filling dead space, enhancing vascularity of the healing wound, and distributing pressure off the bone
Volume 4, Suppl. 1 Nov 2012
Publisher: Kermanshah University of Medical Sciences
URL: http://www.jivresearch.org
Copyright:
Congress of Iranian Neurosurgeons, 14-16 November 2012, Kermanshah, Iran
- Resurfacing with a large regional pedicle flap, with suture line away from the area of direct pressure, and one that
does not encroach on adjacent flap territories
- Preserving options for future potential break-downs
Keyword:
Prevention, Pressure ulcers, Spinal Cord Injury
* Corresponding Author at:
Hmid Reza Saeidi Borojeni: Associate professor of Neurosurgery, Department of Neurosurgery, Kermanshah University of Medical Sciences,
Kermanshah, Iran. Tel: 08318367989, Email: dr_hamidrezasaeidi@yahoo.com, (Saeidi Borogeni HR.).

				

